# Low-Frequency Components of the Heart Sound Corresponding to the Fourth Heart Sound Phase, Assessed by Phonocardiography, Correlate with Early Variations in Echocardiographic Indices Related to Diastolic Function

**DOI:** 10.3390/medicina62071300

**Published:** 2026-07-06

**Authors:** Kunimasa Yagi, Yuhei Yasui, Shumpei Saito, Makoto Iwazawa, Shimpei Ogawa, Taketsugu Tsuchiya, Tomoya Kaneda, Masaji Miyamoto, Fuminori Yamagishi, Junji Kobayashi, Hideki Origasa, Tatsuya Kawasaki, Naohito Yamasaki, Takashi Muro, Nobuo Fukuda

**Affiliations:** 1 Department of Internal Medicine, Kanazawa Medical University Hospital, 1-1 Daigaku, Uchinada, Kahoku 920-0293, Ishikawa, Japan; 2 AMI Inc., 2-13 302 Higashisengokucho, Kagoshima 892-0842, Kagoshima, Japan; yasui.yuhei@ami.inc (Y.Y.); saito@ami.inc (S.S.); makoto.iwazawa@ami.inc (M.I.); sogawa@ami.inc (S.O.); 3 Department of Cardiology, Kanazawa Medical University Hospital, Kahoku 920-0293, Ishikawa, Japan; tsugu@quartz.ocn.ne.jp; 4 Department of Internal Medicine, Keiju Medical Center, 94 Tomioka-cho, Nanao 926-8605, Ishikawa, Japan; tomoya.kaneda@keiju.co.jp (T.K.); masaji.miyamoto@keiju.co.jp (M.M.); 5 Department of Internal Medicine, Itoigawa General Hospital, 457-1 Takegahana, Itoigawa 941-8502, Niigata, Japan; yamafumiicloud@me.com; 6 Department of Medicine, Division of Diabetes, Metabolism and Endocrinology, Chiba University Hospital, Chiba 260-8677, Chiba, Japan; junjimaryland@gmail.com; 7 Data Science and AI Innovation Research Promotion Center, Institute of Statistical Mathematics, Shiga University, Hikone 525-8522, Shiga, Japan; origasahideki@gmail.com; 8 Department of Cardiology, Matsushita Memorial Hospital, Moriguchi 570-8540, Osaka, Japan; tk20140531@gmail.com; 9 Department of Cardiology and Geriatrics, Kochi Medical School, Nankoku 783-8505, Kochi, Japan; yamasakn@kochi-u.ac.jp; 10 Heart Valve Center, Midori Hospital, 1-16 Sumiyoshi, Nishi-ku, Kobe 651-2133, Hyogo, Japan; muroutakashi@gmail.com; 11 Department of Internal Medicine, National Hospital Organization, Shikoku Medical Center for Children and Adults (SMCCA), Zentsuji 765-8507, Kagawa, Japan; nfukuda1949@gmail.com

**Keywords:** heart sounds, phonocardiography, diastolic dysfunction, echocardiography, HFpEF

## Abstract

*Background and Objectives*: The fourth heart sound (S4) is a recognized marker of left ventricular (LV) diastolic dysfunction, suggesting a potential risk of congestive heart failure (CHF). However, low-frequency sounds are often audible during the S4-corresponding phase in the general population without symptoms or a history of CHF. The present retrospective cross-sectional exploratory study examined the acoustic features of the S4 phase sound in patients with stage A or B heart failure in association with echocardiographic markers. *Materials and Methods*: Sixty asymptomatic male patients underwent simultaneous phonocardiographic and electrocardiographic recordings using Cardio-EGG (AMI Inc.). The amplitude of acoustic signals during the S4 phase was quantified across ten frequency bands using a continuous wavelet transform. Echocardiographic parameters of LV diastolic function were also assessed. *Results*: Several lower-frequency bands demonstrated significant correlations with LV diastolic indices. After adjustment for age, systolic blood pressure, HbA1c, interventricular septal thickness, and coronary artery disease, strong associations were observed at the fourth left sternal border signals in the [7.81, 15.63) Hz band and septal E/e′ (r = 0.41, *p* = 0.0012). The [15.63, 31.25) Hz band was associated with septal E/e′, septal and lateral e′ velocities, and the E/A ratio. This frequency range at the apex also correlated with A-wave velocity (*p* = 0.0011). *Conclusions*: Specific low-frequency acoustic components observed during the S4 phase are closely associated with echocardiographic markers of left ventricular diastolic function in asymptomatic individuals. These characteristics resemble those of the standard S4, indicating that digital phonocardiography could facilitate the identification of early cardiac dysfunction before the onset of heart failure.

## 1. Introduction

The fourth heart sound (S4) has long been recognized as an indicator of left ventricular (LV) diastolic dysfunction [1,2]. Early S4 appearance is increasingly emphasized in preventive cardiology, as diastolic dysfunction often precedes overt congestive heart failure (CHF), particularly in cases with preserved ejection fraction (HFpEF) [3]. S4 was initially described in the nineteenth century as a presystolic extra heart sound [4,5]. Classic phonocardiographic studies characterized S4 as a low-frequency, low-amplitude sound occurring in late diastole before the first heart sound [2,6]. Experimental and clinical research in the 1970s [7] and 1980s [8] demonstrated that S4 results from atrial contraction against a noncompliant ventricle, producing damped vibrations of the LV wall and adjacent structures, and S4 has been regarded as a hallmark of a stiff ventricle.

However, low-frequency sound is often audible during the S4-corresponding phase in individuals without symptoms or a history of CHF [9]. To date, no study has demonstrated whether the audible low-frequency component during the S4 phase in asymptomatic individuals aligns with the established S4 frequency (approximately 16–35 Hz) [10] or correlates with diastolic parameters. This question remains unresolved, primarily due to the absence of objective methods for assessing heart sounds in asymptomatic patients.

Recent advances in digital auscultation devices may address these questions. High-fidelity electronic stethoscopes and analytical techniques such as the continuous wavelet transform (CWT) enable precise, quantitative evaluation of heart sound intensity within specific frequency bands [11,12]. These technologies facilitate examination of traditional auscultatory findings and may provide a non-invasive approach to monitoring early cardiac changes in clinical and community settings.

This study aimed to clarify the acoustic nature of the S4-phase sound (S4p) and its relationship with echocardiographic indices of LV diastolic function in patients with stage A or B heart failure. The objective was to determine whether modern sound analysis can quantitatively validate S4p as a marker of LV diastolic function from the pre-heart-failure stage, with potential implications for early risk evaluation.

## 2. Materials and Methods

### 2.1. Study Design

This retrospective cross-sectional observational study followed the ethical standards of the institutional review board and the Declaration of Helsinki. The Research Ethics Committee of Kanazawa Medical University Hospital approved the protocol (IRB Nos. I805, C096, and C111). Written informed consent was obtained from all participants, who were also informed of their right to withdraw at any time.

**Figure 1:**
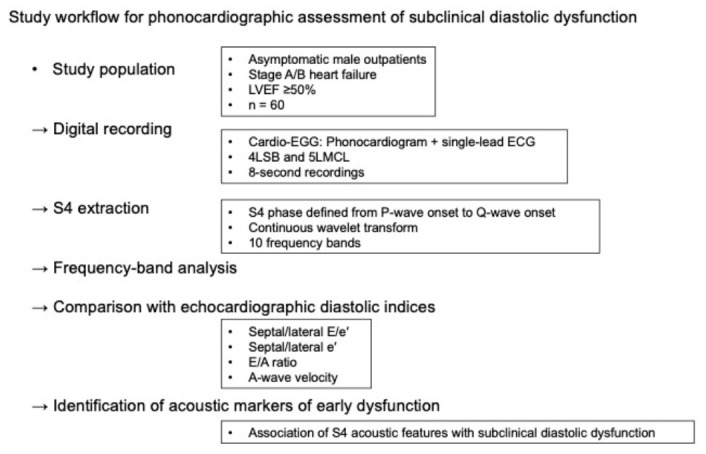
Workflow for phonocardiographic evaluation of subclinical diastolic dysfunction.

The primary outcome of this study was the association between S4p and the septal E/e′ ratio, a key echocardiographic marker of left ventricular filling pressure. The secondary outcome was the association between S4p and additional TTE diastolic indices, including lateral E/e′ ratio, septal e′-velocity, lateral e′-velocity, E/A ratio, E-wave velocity, A-wave velocity, deceleration time (DcT), ejection fraction (EF), and interventricular septum (IVS) value. The study was conducted and reported in accordance with the Strengthening the Reporting of Observational Studies in Epidemiology (STROBE) guidelines. The overall study workflow, including data collection and analysis procedures, is presented in Figure 1.

Asymptomatic male outpatients with early-stage (A/B) heart failure underwent simultaneous phonocardiography and electrocardiography using the Cardio-EGG device (AMI Inc., Kagoshima, Japan). Recordings were obtained at the fourth left sternal border (4LSB) and the fifth left mid-clavicular line (5LMCL), both of which are recognized as standard positions for heart sound assessment. Acoustic signals during the S4 phase were analyzed using a continuous wavelet transform. These signals were examined across predefined ten frequency bands and compared with echocardiographic indices of left ventricular diastolic function.

### 2.2. Study Population

Sixty male outpatients were enrolled from Kanazawa Medical University Hospital, Keiju Medical Center, and Itoigawa General Hospital between November 2023 and October 2024. Inclusion criteria required the availability of data on digital phonocardiography, hemoglobin A1c (HbA1c), plasma B-type natriuretic peptide (BNP), and transthoracic echocardiography (TTE), with an LVEF of at least 50%. Exclusion criteria included symptoms or history of heart failure, genetically confirmed cardiomyopathy, moderate-to-severe valvular disease, persistent atrial fibrillation, percutaneous coronary intervention or transcatheter aortic valve implantation within one year, dialysis dependency, severe renal dysfunction (serum creatinine above 2.0 mg/dL), advanced hepatic impairment (Child-Pugh score 10 or higher), or active malignancy.

### 2.3. Medical Record Review and Variable Definitions

We conducted a comprehensive review of medical examinations, including current and past medical histories and detailed medication records. Type 2 diabetes (T2D) was diagnosed according to the American Diabetes Association criteria [13]. Hypertension was defined as systolic blood pressure (sBP) ≥ 140 mmHg, diastolic blood pressure (dBP) ≥ 90 mmHg, or current use of antihypertensive medications [14]. Dyslipidemia was defined according to the 2017 Guidelines of the Japan Atherosclerosis Society, based on lipid profile thresholds and current lipid-lowering therapy [15]. Proteinuria was diagnosed by urinary albumin excretion of ≥30 mg/dL or a positive result of (±) or higher on urine dipstick testing. Coronary artery disease (CAD) was defined as ≥75% stenosis on coronary angiography or ≥50% stenosis on coronary computed tomography scan within 3 years of enrollment. Plasma BNP levels were measured using a chemiluminescent immunoassay (BNP-JP Abbott reagents; Abbott Japan Co., Ltd., Tokyo, Japan).

### 2.4. Echocardiographic Evaluation

TTE was carried out using a 3.75 MHz probe (EPIQ G7; Philips, Amsterdam, The Netherlands), and standard LV diastolic function parameters were measured, including the E/A ratio, deceleration time, and E/e′ ratio.

Heart failure stage was classified according to the 2025 Guideline of the Japanese Circulation Society and the Japan Heart Failure Society (JCS/JHFS 2025) [16]. Stage B was defined as structural heart disease without signs or symptoms of heart failure, including any of the following: EF <50% (none in this cohort), IVS >12 mm, left atrial diameter (LAD) > 40 mm, LV diastolic diameter (LVDd) > 52 mm, elevated E/e′, decreased e′, or BNP > 35 pg/mL.

LV diastolic dysfunction and its grading were examined in accordance with the 2016 American Society of Echocardiography (ASE) recommendations for patients with LVEF ≥ 50%. LV diastolic dysfunction was diagnosed when at least two of the following were present: elevated E/e′ ratio (average > 14, lateral > 13, or septal > 15), reduced e′ velocity (septal < 7 cm/s or lateral < 10 cm/s), or tricuspid regurgitation velocity (TRV) > 2.8 m/s [17]. A threshold of >8 for septal E/e′ was selected as an exploratory endpoint, as it closely approximates the upper limit of the normal range described in the ASE recommendations. This value does not represent an established diagnostic cutoff for elevated filling pressure or definite diastolic dysfunction. Grade II (elevated LV filling pressure) was assigned when both parameters were abnormal, Grade I when both were normal, and cases with discordant findings were classified as inconclusive.

### 2.5. Digital Phonocardiography

**Figure 2:**
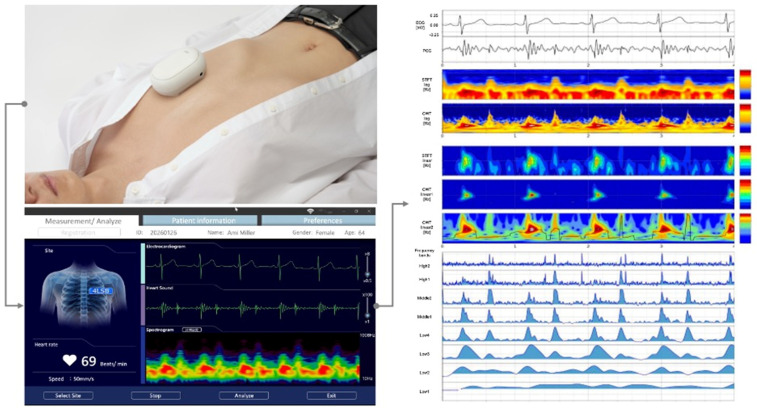
Graphical abstract reproduced with permission from Ref. [11]. 2024, Ogawa S. et al. Workflow: (1) The Cardio-EGG device records heart sounds in a clinical setting (**upper left**). (2) The Cardio-EGG interface displays the acquired phonocardiographic signals (**lower left**). (3) The recorded heart sounds are transmitted via a cloud-based auscultation system (“Cloud Choshin”) and processed using signal evaluation methods, such as continuous wavelet transformation, for AI-assisted diagnostic evaluation (**right**).

The Cardio-EGG (AMI Inc., Kagoshima, Japan) simultaneously records single-lead electrocardiogram (ECG) and phonocardiographic signals (Figure 2) [11]. The reproducibility and reliability of Cardio-EGG are ensured through approval by the Pharmaceuticals and Medical Devices Agency (PMDA) in Japan. Phonocardiographic recordings were conducted under standardized conditions. The room door was closed to minimize background noise. Each subject lay supine and held their breath for eight seconds at the end of exhalation, after several deep breaths. A skilled operator positioned the device on the subject’s chest wall to obtain the recordings. Heart sounds were acquired from a single record at the fourth left sternal border (4LSB) and the fifth intercostal space at the mid-clavicular line (5LMCL). Time-frequency analyses, employing the short-time Fourier transform and CWT on the cloud-based Cloud Choshin service (Supplementary Figure S1), generated visualized phonocardiograms. S4 phase amplitude-based features were extracted for each predefined frequency band. Identification of the S4p was achieved by temporally aligning the phonocardiographic waveform with the P- and Q-wave onsets on the ECG, which correspond to the onset of late diastole. In this study, we excluded paced rhythms and frequent ectopy. We did not exclude prolonged PR intervals or bundle branch blocks, as the context for A-wave and S4 occurrence remained consistent, with no beat-to-beat variations during data collection. S4-phase segmentation was performed automatically. During the development of the automated analysis system, we conducted 200 manual verifications prior to implementation. The intensity of the acoustic signal was normalized relative to the intensity of the first heart sound. Following isolation of the S4 segment, the waveform was decomposed into ten frequency bands using octave-order frequency scaling: level 0: [1.95, 3.91) Hz; level 1: [3.91, 7.81) Hz; level 2: [7.81, 15.63) Hz; level 3: [15.63, 31.25) Hz; level 4: [31.25, 62.5) Hz; level 5: [62.5, 125) Hz; level 6: [125, 250) Hz; level 7: [250, 500) Hz; level 8: [500, 1000) Hz; level 9: [1000, 2000) Hz. The ten-band configuration in the WaveletVisualizer provides a balance between detailed analysis and clinical practicality.

### 2.6. Statistical Analysis

Sample size was calculated to detect a 0.5 difference in the correlation coefficient, based on preliminary data indicating a moderate-to-strong correlation. Statistical power was set at 90% with a two-sided significance level of 1%, resulting in a required sample size of 56. Continuous variables were summarized as means and standard deviations, while categorical variables were presented as frequencies and percentages. Group comparisons were conducted using unpaired t-tests or Mann–Whitney U tests for continuous variables, and chi-squared tests with Yates’ correction for categorical variables, as appropriate.

After conducting univariate analyses, multivariate analyses were performed, adjusting for age, sBP, CAD, HbA1c, and IVS, to examine whether statistically significant predictive models could be developed using multiple linear and logistic regression. These variables were selected a priori based on their known clinical relevance to diastolic function [18,19,20]. Linear regression was used to predict continuous outcomes, whereas logistic regression was used to predict categorical outcomes. These analyses examined the predictive value of S4-related acoustic features for TTE diastolic parameters.

Following multivariate regression analyses, receiver operating characteristic (ROC) analyses were conducted to examine the discriminative capacity of selected S4 acoustic features for predefined echocardiographic abnormalities. The area under the curve (AUC) and corresponding 95% confidence intervals (CI) were calculated. Optimal cut-off values were identified using the Youden index. These analyses were exploratory in nature.

Given the exploratory design of this study, no formal correction for multiple comparisons was implemented; however, we examined the false discovery rate (FDR) for items with *p* < 0.005. Each frequency band was examined independently and may possess potential physiological relevance. Further validation in independent cohorts is warranted.

Missing data were minimal, typically 1–2 cases per variable. A complete case analysis was performed. Observations with missing values were excluded from the corresponding analyses, and no imputation was applied.

All statistical analyses were conducted using R version 4.3.0 with RStudio version 2024.09.1 + 394 (R Foundation for Statistical Computing, Vienna, Austria) and JMP Student Edition version 18.2.0 (SAS Institute Inc., Cary, NC, USA). Analyses were performed on a Macintosh computer.

## 3. Results

Table 1 summarizes the participants’ clinical characteristics. The mean age was 64.4 ± 16.7 years, and the mean BMI was 25.3 ± 4.4 kg/m^2^. Definite LV diastolic dysfunction was observed in just one participant, while six were inconclusive. Fifteen patients (25%) were classified as Stage A and 45 (75%) as Stage B, based on the JCS/JHFS 2025 Guideline.

Several low-frequency bands of S4p showed significant correlations with TTE indices of LV diastolic function. FDR-q values were assessed for subjects with *p*-values less than 0.05. Among the associations between echocardiographic functional parameters (dependent variables) and heart sound levels (independent variables), 11 remained statistically significant following FDR-q evaluations. For example, signals in the 4LSB level 2 [7.81, 15.63) Hz band were associated with septal E/e′ (R^2^ = 0.17, r = 0.41, *p* = 0.0012, FDR-q = 0.035). The septal E/e′ ratio demonstrated significant associations with the 2–3 [7.81, 31.25) Hz frequency bands at 4LSB (Table 2).

Multivariate regression analysis demonstrated that the 4LSB level 2 [7.81, 15.63) Hz band was associated with septal E/e′ (β = 0.73, 95% CI: 0.13–1.34, *p* = 0.0019) (Supplementary Table S1). Notably, 4LSB level 2 [7.81, 15.63) Hz yielded a higher coefficient of determination (R^2^ = 0.39), suggesting greater predictive value. Furthermore, 4LSB level 3 [15.63, 31.25) Hz was significantly associated with lateral E/e′, septal e′, lateral e′, and the E/A ratio. The 5LMCL level 3 [15.63, 31.25) Hz band was associated with A-wave velocity (β = 13.56, 95% CI: 3.98–23.15, *p* = 0.0060).

Furthermore, the associations between each S4p frequency band and established ASE cutoff values for LV diastolic dysfunction were examined (Table 3 and Supplementary Table S2). FDR-q values were also assessed for subjects with *p*-values less than 0.05. Among the associations between echocardiographic functional parameters (dependent variables) and heart sound levels (independent variables), 11 remained statistically significant following FDR-q evaluations. 4LSB level 2 was significantly associated with septal E/e′ ≤ 8, septal e’, and lateral e′ < 10 cm/s. Additionally, 4LSB level 3 was significantly associated with septal e′ ≤ 7 cm/s.

**Figure 3:**
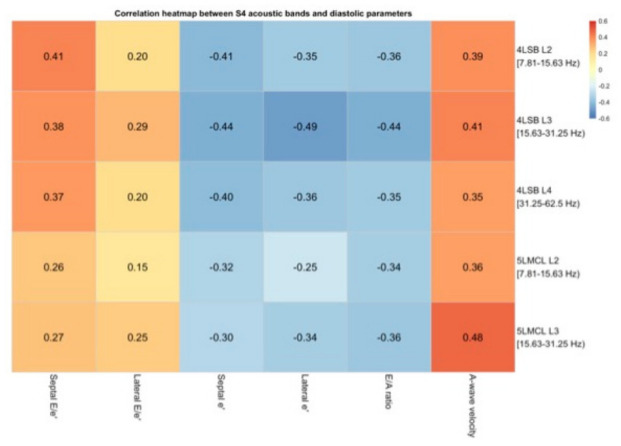
Correlation heatmap illustrating associations between S4 acoustic bands and diastolic parameters from transthoracic echocardiography (TTE).

**Figure 4:**
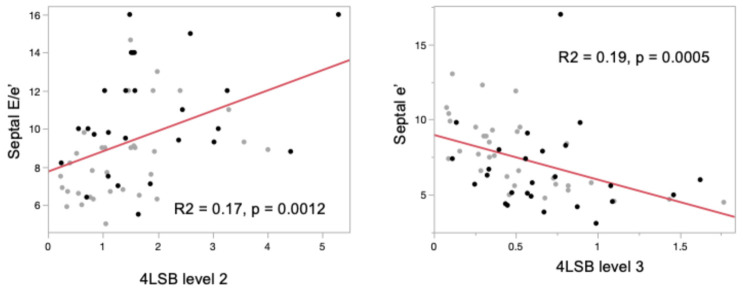
Scatter plots for the principal associations, especially 4LSB level 2 vs. septal E/e′ and 4LSB level 3 vs. e’ velocities.

Low-frequency acoustic signals of S4p, specifically from 4LSB levels 2 and 3, demonstrated strong and independent associations with both continuous and categorical TTE markers of LV diastolic dysfunction in subjects with stage A and B heart failure (Figure 3 and Figure 4). These signals were reflected in frequency bands overlapping the classic S4 range.

The heatmap presents significant associations identified in Table 1. Orange-to-yellow columns indicate models in which the heart-sound-level area contributed positively, with deeper orange signifying a stronger contribution. Values represent model correlation coefficients (r). 4LSB, fourth left sternal border; 5LMCL, fifth left midclavicular line; E/A, ratio of early to atrial transmitral flow velocities; E/e′, ratio of E-wave velocity to annular e′ by tissue Doppler; TTE, transthoracic echocardiography.

Exploratory ROC analyses assessed whether S4 acoustic features distinguish threshold-defined abnormalities in diastolic indices (Supplementary Figure S2), with septal E/e′ > 8 used as an exploratory endpoint representing an early deviation from the normal range, rather than an established diagnostic threshold for elevated LV filling pressure. S4 amplitude at the 4LSB level 2 (7.81–15.63 Hz) exhibited moderate discriminative ability, as reflected by an AUC of 0.73 (95% CI, 0.60–0.86). The optimal cut-off value provided a sensitivity of 76.2% and a specificity of 71.0%. These results suggest that low-frequency S4 amplitude components may help identify early diastolic abnormalities with external validation. Although these analyses were exploratory and not prespecified, they indicate the potential utility of S4 acoustic features for non-invasive evaluation of subclinical diastolic dysfunction.

## 4. Discussion

This study revealed that S4p was similar to regular S4. A distinctive aspect of the present study is that heart sound evaluation was conducted in pre–heart failure patients without CHF symptoms and history, and without conditions such as definite aortic stenosis or hypertrophic cardiomyopathy with genetic backgrounds. The principal frequency components were identified between 7.81 and 31.25 Hz, aligning with the established low-frequency range for S4 (approximately 16–35 Hz) [10,17].

The consistent pattern observed in this cohort indicates that Cardio-EGG reliably quantifies subtle S4-phase acoustic features. The S4 phase is typically subtle and often difficult to detect by conventional auscultation, as evidenced by the low prevalence of guideline-defined left ventricular diastolic dysfunction in the study population. Cardio-EGG further quantified low-frequency acoustic features during the S4 phase that correlated with echocardiographic indices of diastolic function in this predominantly pre-heart-failure population [21].

Distinct S4 phase-frequency bands correspond to specific TTE parameters. The lower-frequency component at the 4LSB level 2 (7.81–15.63 Hz) shows a strong correlation with septal E/e′, an established marker of LV filling pressure, suggesting a potential association with ventricular pressure changes. In contrast, higher-frequency bands at 4LSB level 3 (15.63–31.25 Hz) are correlated with septal E/e′ ratio and e′ velocities, which serve as indicators of myocardial relaxation. At the apex, comparable frequency bands are associated with A-wave velocity, reflecting atrial contraction. Notably, these associations remained robust after adjustment for clinical variables, including age, sBP, glycemic status, wall thickness, and CAD. Thus, analysis of these frequency bands may facilitate the characterization of diastolic dysfunction and improve risk stratification [22,23,24], including in asymptomatic individuals. However, because these associations were identified from analyses across multiple acoustic frequency bands and echocardiographic endpoints, they should be considered exploratory. While the FDR analyses supported the robustness of the principal associations, external validation is necessary before clinical implementation. Furthermore, because definitive left ventricular diastolic dysfunction was uncommon in this cohort, ROC analyses employed an exploratory early-variation endpoint (septal E/e′ >8), which approximates the upper limit of the normal range described in current echocardiographic guidelines, rather than an established diagnostic threshold for elevated filling pressure. Therefore, these ROC findings should be viewed as hypothesis-generating rather than as a foundation for clinical application.

An unexpected finding was that S4p at 4LSB demonstrated greater sensitivity than recordings obtained from the apex. In conventional phonocardiography, S4 at the heart’s apex typically exhibits the highest amplitude. This pattern may be attributed to increased stretching along the heart’s longitudinal axis during atrial contraction [25]. Apical recordings capture more of this longitudinal motion and contain higher-frequency components that correspond to the A-wave velocity. In contrast, 4LSB recordings primarily reflect short-axis wall motion, capturing lower-frequency components that are more closely associated with left ventricular relaxation and filling pressure.

Digital phonocardiography, unlike TTE, can be performed rapidly and repeatedly, enabling longitudinal monitoring of cardiac function. This technique requires minimal equipment and time and does not necessitate specialized expertise. When combined with signal processing and AI, digital phonocardiography offers a simple, scalable, and cost-effective approach to cardiac assessment [26]. It is also suitable for evaluating early cardiac abnormalities, particularly in asymptomatic individuals at elevated risk. Further research involving individuals with stage B heart disease is warranted to expand the evidence base. If these advantages are validated, digital phonocardiography may be integrated into routine clinical practice, community-based examinations, and remote health monitoring, providing a simple, non-invasive approach to monitoring S4-phase acoustic features associated with echocardiographic indices of diastolic function before the onset of heart failure [27]. Ongoing advancements in AI are expected to further enhance the accuracy of this method [12].

The primary limitation of this study is the exclusive inclusion of male participants, which substantially restricts the external validity of the findingsd. The cohort was limited to men to minimize sex-related physiological variability in diastolic function among middle-aged and older adults [28]. These results should be interpreted as primarily applicable to male patients and should be validated in independent female cohorts prior to broader clinical application. A cross-sectional design limits causal inferences. The lack of formal assessment of inter- and intra-observer variability in signal collection and processing may impact reproducibility. Given the relatively small sample size, the risk of overfitting cannot be ruled out; nevertheless, as this was designed as a pilot study, the observed associations may serve as hypotheses for future validation studies. Furthermore, the examination of multiple frequency bands, recording sites, echocardiographic parameters, and threshold-defined categorical endpoints introduces the possibility of false-positive findings due to multiple testing. Although FDR analyses supported the robustness of the principal associations, these findings should still be interpreted with caution until validated in independent cohorts. Despite these limitations, the consistency of the principal associations across different TTE parameters and recording sites provides some support for the robustness of the observed findings. Finally, the echocardiographic assessment was performed according to the 2016 ASE recommendations, whereas updated recommendations became available in 2025 [29]. Because left atrial reservoir strain (LARS), newly incorporated into the updated recommendations [30], was not routinely measured during the study period. Accordingly, the analyses were performed using the 2016 ASE recommendations.

## 5. Conclusions

In conclusion, low-frequency acoustic components during the S4 phase, particularly those recorded at 4LSB, were associated with TTE indices of left LV diastolic function. The findings indicate that modern digital phonocardiography may complement conventional imaging in assessing early LV diastolic abnormalities and be valuable for longitudinal monitoring in at-risk populations. As this exploratory study included only male participants, these results should be interpreted with cautioen before generalization to the broader population.

## Figures and Tables

**Table 1:** Baseline Clinical Characteristics of the Study Population.

Characteristics	Missing (N)	N = 60
age, years old	0	64.4 ± 16.7
T2D, (%)	0	25 (42%)
CAD, (%)	0	12 (20%)
HT (%)	0	40 (67%)
DL (%)	0	54 (90%)
BMI	0	25.3 ± 4.4
sBP, mmHg	0	127.4 ± 13.5
dBP, mmHg	0	73.8 ± 11.5
HR	0	70.6 ±13.4
HbA1c, %	0	6.16 ± 0.71
PG, mg/dL	0	120.7 ± 33.9
Hb, g/dL	0	14.6 ± 1.5
Ht, %	0	44.0 ± 4.3
TC, mg/dL	0	183.8 ± 34.6
TG, mg/dL	0	132.5 ± 73.6
LDL-C, mg/dL	0	104.7 ± 32.9
HDL-C, mg/dL	0	58.6 ± 17.4
Cre, mg/dL	0	0.90 ± 0.17
eGFR, mL/min/1.73m^2^	0	69.7 ± 15.3
UA, mg/dL	0	5.8 ± 1.0
ALT, IU/L	0	30.5 ± 24.0
γ-GTP	0	38.4 ± 33.0
UAE, mg/gCre	0	33.8 ± 78.7
BNP, ng/mL	0	20.2 ± 34.5
BNP >35 ng/mL	0	8 (14%)
heart failure stage ([16])	0	
stage A		15 (25%)
stage B		45 (75%)
BNP > 35		8
IVS ≥ 12		4
LVDd ≥ 52		5
LAD ≥ 40		14
elevated E/e′		6
reduced e’		20
stage C&D		0 (0%)
SGLT2i (%)	0	0 (0%)
insulin (%)	0	1 (2%)
GLP-1a (%)	0	3 (5%)
thiazide use	0	3 (5%)
TTE parameters		
LAD, mm	0	37.4 ± 4.7
LVDd, mm	0	47.2 ± 3.3
IVS, mm	0	9.4 ± 1.4
EF, %	0	62.5 ± 5.5
septal E/e′	1	9.4 ± 2.8
lateral E/e′	1	7.9 ± 2.9
average E/e′	1	8.6 ± 2.7
septal e’, m/s	1	7.2 ± 2.6
lateral e’, m/s	1	8.7 ± 3.2
E, m/s	0	64.3 ± 14.3
A, m/s	0	75.0 ± 17.7
E/A	0	0.91 ± 0.36
TRV, m/s	21	1.50 ± 1.13
DcT, ms	1	209.7 ± 50.5

Continuous variables are presented as mean ± standard deviation (SD), and categorical variables as number (%). Heart failure stage was classified according to JCS 2025 guideline. Grading of LV diastolic dysfunction was performed in accordance with ASE 2016 recommendations. Abbreviations: ALT, alanine aminotransferase; ASE, American Society of Echocardiography; BMI, body mass index; BNP, brain natriuretic peptide; CAD, coronary artery disease; Cre, creatinine; dBP, diastolic blood pressure; DcT, deceleration time; DL, dyslipidemia; E/A, ratio of peak early diastolic (E) to peak atrial systolic (A) transmitral flow velocities; ECG, electrocardiography; E/e′, ratio of early diastolic transmitral flow velocity to early diastolic mitral annular velocity by tissue Doppler imaging; EF, left ventricular ejection fraction; eGFR, estimated glomerular filtration rate; Hb, hemoglobin; HDL-C, high-density lipoprotein cholesterol; HT, hypertension; IVS, interventricular septal thickness; JCS, The Japanese Circulation Society; LAD, left atrial diameter; LDL-C, low-density lipoprotein cholesterol; LV, left ventricle; LVDd, left ventricular end-diastolic diameter; PG, plasma glucose; sBP, systolic blood pressure; T2D, type 2 diabetes; TC, total cholesterol; TG, triglyceride; TRV, tricuspid regurgitation velocity; TTE, transthoracic echocardiography; UA, uric acid; UAE, urinary albumin excretion.

**Table 2:** Univariate Linear Regression Analysis Between S4-Phase Acoustic Signal Intensity and Echocardiographic Parameters (Continuous Variables).

**(a) at 4LSB**																										
		**Septal E/e′**			**Lateral E/e′**		**Septal e′**			**Lateral e′**			**E/A**			**E**		**A Wave**			**DcT**		**EF**		**IVS**	
	**Frequency Bands**	**R2**	***p***	**FDR_q**	**R2**	***p***	**R2**	***p***	**FDR_q**	**R2**	***p***	**FDR_q**	**R2**	***p***	**FDR_q**	**R2**	***p***	**R2**	***p***	**FDR_q**	**R2**	***p***	**R2**	***p***	**R2**	***p***
level_0_area	[1.95, 3.91) Hz	0.011	0.43		0.0038	0.65	0.022	0.27		0.023	0.25		0.013	0.40		0.003	0.69	0.0057	0.57		0.0010	0.81	0.000023	0.97	0.012	0.41
level_1_area	[3.91, 7.81) Hz	0.030	0.19		0.018	0.31	0.040	0.13		0.054	0.079		0.045	0.11		0.0082	0.49	0.060	0.061		0.0025	0.71	0.012	0.40	0.0056	0.57
level_2_area	[7.81, 15.63) Hz	0.17	0.0012	0.035	0.039	0.14	0.17	0.0012	0.035	0.12	0.0074	0.067	0.13	0.0054	0.060	0.023	0.25	0.15	0.0024	0.049	0.051	0.087	0.0024	0.71	0.0257	0.22
level_3_area	[15.63, 31.25) Hz	0.15	0.0027	0.049	0.084	0.028	0.19	0.00052	0.026	0.24	0.00011	0.013	0.20	0.00043	0.026	0.060	0.06	0.17	0.0011	0.035	0.038	0.14	0.0080	0.50	0.018	0.31
level_4_area	[31.25, 62.5) Hz	0.13	0.0045	0.059	0.041	0.13	0.16	0.0017	0.037	0.13	0.0061	0.061	0.12	0.0066	0.063	0.030	0.19	0.12	0.0060	0.061	0.022	0.27	0.0053	0.58	0.0055	0.57
level_5_area	[62.5, 125) Hz	0.021	0.27		0.00091	0.82	0.060	0.062		0.037	0.15		0.059	0.063		0.023	0.24	0.038	0.14		0.014	0.37	0.028	0.21	0.0076	0.51
level_6_area	[125, 250) Hz	0.045	0.11		0.013	0.40	0.059	0.063		0.028	0.21		0.020	0.29		0.0040	0.63	0.024	0.24		0.0010	0.81	0.062	0.055	0.00049	0.87
level_7_area	[250, 500) Hz	0.0036	0.65		0.00040	0.88	0.00094	0.82		0.00050	0.87		0.00060	0.85		0.0059	0.56	0.00056	0.86		0.00026	0.90	0.041	0.12	0.0014	0.77
level_8_area	[500, 1000) Hz	0.033	0.17		0.0071	0.53	0.039	0.13		0.017	0.32		0.012	0.42		0.00010	0.94	0.010	0.46		0.0011	0.81	0.031	0.18	0.00056	0.86
level_9_area	[1000, 2000) Hz	0.019	0.30		0.010	0.45	0.027	0.21		0.027	0.22		0.013	0.38		0.0024	0.71	0.0082	0.50		0.00065	0.85	0.0044	0.62	0.000050	0.96
**(b) at 5LMCL**																										
		**Septal E/e′**			**Lateral E/e′**		**Septal e′**			**Lateral e′**			**E/A**			**E**		**A Wave**			**DcT**		**EF**		**IVS**	
	**Frequency Bands**	**R2**	***p***		**R2**	***p***	**R2**	***p***	**FDR_q**	**R2**	***p***	**FDR_q**	**R2**	***p***		**R2**	***p***	**R2**	***p***	**FDR_q**	**R2**	***p***	**R2**	***p***	**R2**	***p***
level_0_area	[1.95, 3.91) Hz	0.038	0.14		0.028	0.21	0.16	0.0015	0.037	0.13	0.0047	0.059	0.080	0.030		0.097	0.015	0.0043	0.62		0.0031	0.68	0.046	0.10	0.0091	0.47
level_1_area	[3.91, 7.81) Hz	0.062	0.057		0.0032	0.67	0.13	0.0049	0.059	0.073	0.041		0.070	0.043		0.074	0.035	0.016	0.33		0.0048	0.60	0.026	0.22	0.011	0.42
level_2_area	[7.81, 15.63) Hz	0.066	0.049		0.021	0.28	0.10	0.013		0.062	0.060		0.11	0.0087		0.034	0.16	0.13	0.0050	0.059	0.012	0.42	0.00025	0.90	0.024	0.24
level_3_area	[15.63, 31.25) Hz	0.075	0.036		0.064	0.056	0.090	0.021		0.12	0.0091		0.13	0.0050	0.059	0.017	0.32	0.23	0.00013	0.013	0.022	0.27	0.022	0.26	0.0017	0.75
level_4_area	[31.25, 62.5) Hz	0.11	0.012		0.084	0.027	0.11	0.011		0.11	0.012		0.10	0.012		0.0080	0.50	0.14	0.0035	0.058	0.055	0.08	0.053	0.076	0.00000030	1.00
level_5_area	[62.5, 125) Hz	0.016	0.35		0.000039	0.96	0.027	0.21		0.0046	0.61		0.015	0.35		0.011	0.43	0.0054	0.58		0.0072	0.53	0.057	0.065	0.011	0.43
level_6_area	[125, 250) Hz	0.021	0.27		0.00079	0.83	0.040	0.13		0.015	0.37		0.00011	0.94		0.012	0.41	0.020	0.29		0.018	0.32	0.015	0.35	0.0016	0.76
level_7_area	[250, 500) Hz	0.015	0.35		0.0043	0.62	0.00029	0.90		0.0014	0.78		0.027	0.22		0.010	0.45	0.031	0.18		0.050	0.091	0.0034	0.66	0.0067	0.53
level_8_area	[500, 1000) Hz	0.0075	0.51		0.00020	0.92	0.016	0.34		0.0032	0.67		0.000067	0.95		0.010	0.45	0.021	0.28		0.053	0.082	0.041	0.12	0.013	0.38
level_9_area	[1000, 2000) Hz	0.0027	0.70		0.0079	0.51	0.017	0.32		0.00068	0.85		0.0013	0.79		0.023	0.25	0.014	0.37		0.060	0.064	0.090	0.020	0.015	0.36

Univariate linear regression analyses were performed with heart sound level area as the independent variable and transthoracic echocardiographic (TTE) parameters as the dependent variables for each frequency band at each recording site. Columns indicate frequency bands in which heart sound level area was confirmed to contribute significantly in the multivariable models shown in Supplementary Table S1. Given the exploratory design of this study, no formal correction for multiple comparisons was implemented; however, we examined the false discovery rate (FDR) for items with *p* < 0.005. 4LSB, fourth left sternal border; 5LMCL, fifth left midclavicular line; DcT, deceleration time; E/A, ratio of peak early diastolic (E) to peak atrial systolic (A) transmitral flow velocities; E/e′, ratio of transmitral E-wave velocity to mitral annular e′ velocity measured by tissue Doppler imaging; EF, left ventricular ejection fraction; IVS, interventricular septal thickness; TTE, transthoracic echocardiography.

**Table 3:** Univariate Logistic Regression Analysis Between S4-Phase Acoustic Signal Intensity and Echocardiographic Parameters Using Guideline-Based Cutoff Values.

**(a) at 4LSB**																	
		**Septal E/e′ ≥ 8 (N = 38)**			**Lateral E/e′ ≥ 10 (N = 8)**		**Septal e′< 7 (N = 31)**			**Lateral e′ < 10 (N = 39)**			**E/A > 0.8 (N = 31)**			**IVS ≥ 10 (N = 27)**	
	**Frequency Bands**	**R2**	***p***	**FDR_q**	**R2**	***p***	**R2**	***p***	**FDR_q**	**R2**	***p***	**FDR_q**	**R2**	***p***	**FDR_q**	**R2**	***p***
level_0_area	[1.95, 3.91) Hz	0.029	0.20		0.00012	0.93	0.026	0.22		0.057	0.070		0.042	0.122		0.023	0.25
level_1_area	[3.91, 7.81) Hz	0.033	0.17		0.0020	0.74	0.065	0.051		0.074	0.039		0.093	0.019		0.030	0.19
level_2_area	[7.81, 15.63) Hz	0.15	0.0022	0.038	0.0010	0.81	0.24	<0.0001	0.006	0.151	0.0026	0.039	0.17	0.0010	0.024	0.053	0.078
level_3_area	[15.63, 31.25) Hz	0.091	0.020		0.042	0.12	0.26	<0.0001	0.006	0.14	0.0040	0.046	0.17	0.0011	0.024	0.039	0.13
level_4_area	[31.25, 62.5) Hz	0.092	0.020		0.024	0.25	0.18	0.00089	0.024	0.11	0.010		0.17	0.0012	0.024	0.033	0.16
level_5_area	[62.5, 125) Hz	0.043	0.11		0.00053	0.86	0.044	0.11		0.036	0.156		0.050	0.088		0.00021	0.91
level_6_area	[125, 250) Hz	0.061	0.060		0.0047	0.61	0.058	0.066		0.020	0.293		0.056	0.070		0.0017	0.75
level_7_area	[250, 500) Hz	0.031	0.19		0.0079	0.51	0.00025	0.91		0.0062	0.557		0.0017	0.76		0.014	0.36
level_8_area	[500, 1000) Hz	0.084	0.026		0.0019	0.75	0.020	0.29		0.047	0.101		0.0016	0.76		0.00051	0.86
level_9_area	[1000, 2000) Hz	0.027	0.21		0.017	0.32	0.022	0.26		0.033	0.174		0.0065	0.54		0.0069	0.53
**(b) at 5LMCL**																	
		**Septal E/e′ ≥ 8 (N = 38)**			**Lateral E/e′ ≥ 10 (N = 8)**		**Septal e′< 7 (N = 31)**			**Lateral e′ < 10 (N = 39)**			**E/A > 0.8 (N = 31)**			**IVS ≥ 10 (N = 27)**	
	**Frequency Bands**	**R2**	***p***		**R2**	***p***	**R2**	***p***		**R2**	***p***		**R2**	***p***	**FDR_q**	**R2**	***p***
level_0_area	[1.95, 3.91) Hz	0.083	0.027		0.017	0.33	0.13	0.0058		0.10	0.014		0.049	0.090		0.022	0.26
level_1_area	[3.91, 7.81) Hz	0.060	0.063		0.0039	0.64	0.096	0.017		0.065	0.054		0.063	0.056		0.061	0.056
level_2_area	[7.81, 15.63) Hz	0.020	0.28		0.033	0.17	0.071	0.041		0.041	0.127		0.14	0.0042	0.046	0.079	0.030
level_3_area	[15.63, 31.25) Hz	0.031	0.18		0.016	0.35	0.047	0.099		0.12	0.0066		0.14	0.0041	0.046	0.031	0.18
level_4_area	[31.25, 62.5) Hz	0.053	0.081		0.051	0.090	0.053	0.079		0.070	0.045		0.078	0.033		0.038	0.14
level_5_area	[62.5, 125) Hz	0.018	0.31		0.000093	0.94	0.0049	0.60		0.0023	0.719		0.029	0.20		0.0029	0.68
level_6_area	[125, 250) Hz	0.095	0.017		0.0042	0.63	0.029	0.20		0.019	0.301		0.0050	0.60		0.0095	0.46
level_7_area	[250, 500) Hz	0.057	0.068		0.0012	0.80	0.0088	0.48		0.011	0.443		0.023	0.25		0.0060	0.56
level_8_area	[500, 1000) Hz	0.026	0.22		0.010	0.46	0.012	0.40		0.0088	0.484		0.0058	0.57		0.022	0.25
level_9_area	[1000, 2000) Hz	0.012	0.41		0.018	0.32	0.0082	0.49		0.0030	0.681		0.019	0.30		0.026	0.22

Univariate logistic regression analyses were performed with heart sound level area as the independent variable and dichotomized transthoracic echocardiographic (TTE) parameters defined by guideline-recommended cutoff values as the dependent variables. The coefficient of determination (R^2^) for each model is shown for each frequency band at each recording site.n5). Columnow indicate frequency bands in which heart sound level area was confirmed to contribute significantly in the multivariable models shown in Supplementary Table S2. Given the exploratory design of this study, no formal correction for multiple comparisons was implemented; however, we examined the false discovery rate (FDR) for items with *p* < 0.005. Valueinled indicate the highest R^2^ among the corresponding models. 4LSB, fourth left sternal border; 5LMCL, fifth left midclavicular line; E/A, ratio of peak early diastolic (E) to peak atrial systolic (A) transmitral flow velocities; E/e′, ratio of transmitral E-wave velocity to mitral annular e′ velocity measured by tissue Doppler imaging; IVS, interventricular septal thickness; LAD, left atrial diameter; LVDd, left ventricular diastolic diameter; N, number of the subjects; TTE, transthoracic echocardiography.

## Data Availability

Deidentified individual participant data reported in this article will be shared upon reasonable request. Data will be available beginning three months after publication and for up to five years. Researchers with a methodologically sound proposal supporting an approved study may request access by contacting the corresponding author. Data will be provided following proposal approval and completion of a data use agreement.

## Supplementary Materials

The following supporting information can be downloaded at: https://www.mdpi.com/article/10.3390/medicina62071300/s1, Figure S1: Assessment of the cardiac fourth heart sound utilizing the Cardio-EGG system and the Cloud Choshin platform; Figure S2: Receiver operating characteristic (ROC) curves illustrating the relationship between S4 acoustic features and septal E/e′; Table S1: Multivariable Linear Regression Analysis Between S4-Phase Acoustic Signal Intensity and Echocardiographic Parameters (Continuous Variables); Table S2: Multivariable Logistic Regression Analysis Between S4-Phase Acoustic Signal Intensity and Echocardiographic Parameters Using Guideline-Based Cutoff Values.

## Abbreviations

The following abbreviations are used in this manuscript:

S4	the fourth heart sound
LV	left ventricular
CHF	congestive heart failure
HFpEF	heart failure with preserved ejection fraction
CWT	continuous wavelet transform
S4p	S4-phase sound
DcT	deceleration time
EF	ejection fraction
IVS	interventricular septum
STROBE	Strengthening the Reporting of Observational Studies in Epidem
HbA1c	hemoglobin A1c
BNP	B-type natriuretic peptide
TTE	transthoracic echocardiography
T2D	type 2 diabetes
sBP	systolic blood pressure
dBP	diastolic blood pressure
CAD	coronary artery disease
LAD	left atrial diameter
LVDd	LV diastolic diameter
ASE	American Society of Echocardiography
TRV	tricuspid regurgitation velocity
ECG	electrocardiogram
4LSB	the fourth left sternal border
5MCL	the fifth intercostal space at the mid-clavicular line
ROC	receiver operating characteristic
AUC	area under the curve
CI	confidence intervals
AI	artificial intelligence
LARS	left atrial reservoir strain
