# Supplementary material for: Low-Frequency Components of the Heart Sound Corresponding to the Fourth Heart Sound Phase, Assessed by Phonocardiography, Correlate with Early Variations in Echocardiographic Indices Related to Diastolic Function
Source: Medicina (Kaunas). 2026 Jul 6;62(7):1300. doi: 10.3390/medicina62071300 (PMC13413805; doi:10.3390/medicina62071300)
Supplement: Supplementary file 1 [file medicina-62-01300-s001.zip › Figure S1&S2 07042026.pdf]

Supplementary Figure S1

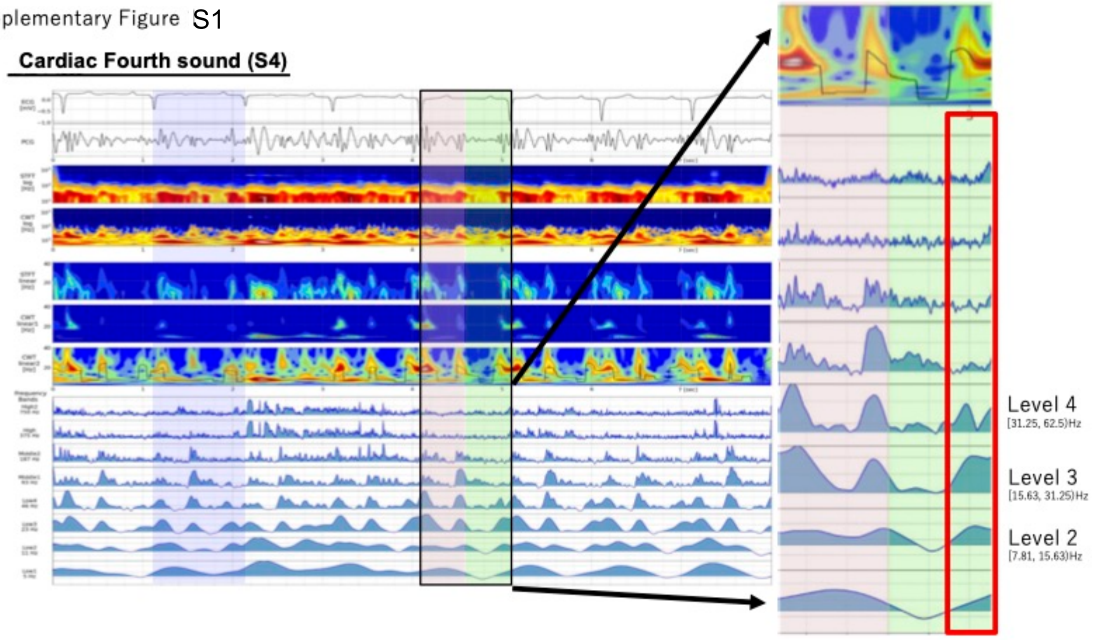


Supplementary Figure S1. Assessment of the cardiac fourth heart sound utilizing the Cardio-EGG system and the Cloud Choshin platform.

The fourth heart sound (S4) was evaluated during the interval from the onset of the P wave, which indicates atrial contraction, to the onset of the Q wave, which marks the beginning of ventricular depolarization, on the electrocardiogram (ECG). An enlarged view of a single cardiac cycle is provided on the right for clarity. Within the red box, the following recordings are displayed from top to bottom: ECG, phonocardiogram, and two representations of the continuous wavelet transform (CWT).

Supplementary Figure S2

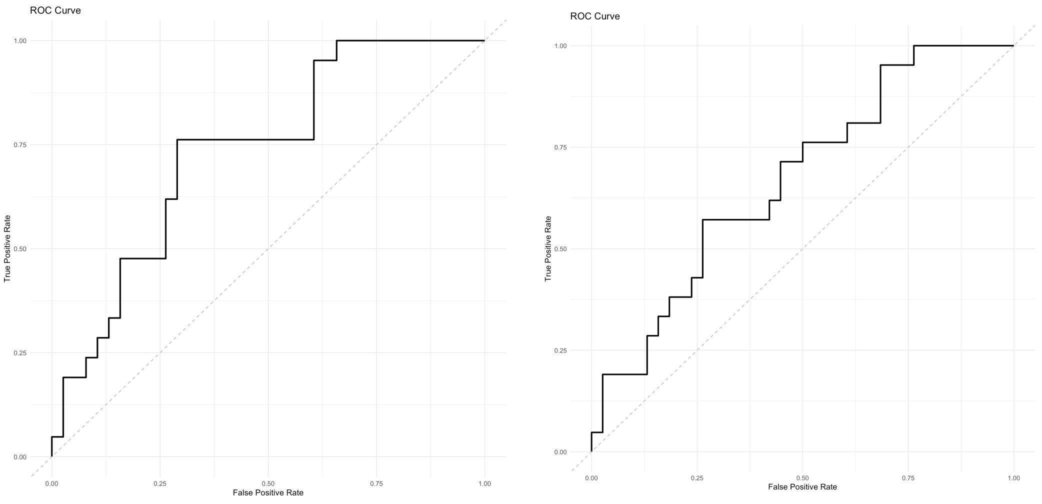


4LSB level 2. Septal E/e'≥8
Area under the curve: 0.73
95% CI: 0.60-0.86 (DeLong)

4LSB level 3 Septal E/e'≥8
Area under the curve: 0.67
95% CI: 0.53-0.81 (DeLong)

Supplementary Figure S2. Receiver operating characteristic (ROC) curves illustrating the relationship between S4 acoustic features and septal E/e′.

The ROC curves illustrate the discriminatory performance of S4 acoustic amplitudes in identifying septal E/e′ values greater than 8. The left panel presents results from the 4LSB level 2 frequency band, while the right panel displays results from the 4LSB level 3 frequency band. The corresponding area under the curve (AUC) values are 0.73 (95% CI, 0.60–0.86) and 0.67 (95% CI, 0.53–0.81).
