# Supplementary material for: Low-Frequency Components of the Heart Sound Corresponding to the Fourth Heart Sound Phase, Assessed by Phonocardiography, Correlate with Early Variations in Echocardiographic Indices Related to Diastolic Function
Source: Medicina (Kaunas). 2026 Jul 6;62(7):1300. doi: 10.3390/medicina62071300 (PMC13413805; doi:10.3390/medicina62071300)
Supplement: Supplementary file 1 [file medicina-62-01300-s001.zip › Table S1 07042026.pdf]

Supplementary Table S1. Multivariable Linear Regression Analysis Between S4-Phase Acoustic Signal Intensity and Echocardiographic Parameters (Continuous Variables)

Independent variable	Dependent variable	β coefficient	SE	p-value
**4LSB level_2_area**	**septal E/e'**	0.733	0.301	0.019
	**septal e'**	-0.561	0.250	0.029
	**A wave**	3.098	1.518	0.047
**4LSB level_3_area**	**septal E/e'**	1.799	0.898	0.051
	**septal e'**	-1.655	0.732	0.028
	**lateral e'**	-2.079	0.800	0.012
	**E/A**	-0.172	0.082	0.041
	**A wave**	6.666	4.534	0.148
**4LSB level_4_area**	**septal e'**	-4.627	2.382	0.058
**5LMCL level_0_area**	**septal e'**	-0.253	0.139	0.074
**5LMCL level_3_area**	**A wave**	13.563	4.771	0.006

Multivariable linear regression analyses were performed with heart sound level area and established contributors to diastolic dysfunction (age, systolic blood pressure, history of CAD, HbA1c, and interventricular septal thickness) as independent variables, and transthoracic echocardiographic (TTE) parameters as dependent variables.

4LSB, fourth left sternal border; 5LMCL, fifth left midclavicular line; AIC, Akaike Information Criterion; CAD, coronary artery disease; DcT, deceleration time; E/A, ratio of peak early diastolic (E) to peak atrial systolic (A) transmitral flow velocities; E/e′, ratio of transmitral E-wave velocity to mitral annular e′ velocity measured by tissue Doppler imaging; EF, left ventricular ejection fraction; F-p-value, the statistic used in the F-test and its significance; IVS, interventricular septal thickness; R2, Nagelkerke R^2; sBP, systolic blood pressure; SE, standard error; TTE, transthoracic echocardiography.
