# Supplementary material for: Low-Frequency Components of the Heart Sound Corresponding to the Fourth Heart Sound Phase, Assessed by Phonocardiography, Correlate with Early Variations in Echocardiographic Indices Related to Diastolic Function
Source: Medicina (Kaunas). 2026 Jul 6;62(7):1300. doi: 10.3390/medicina62071300 (PMC13413805; doi:10.3390/medicina62071300)
Supplement: Supplementary file 1 [file medicina-62-01300-s001.zip › Table S2 07042026.pdf]

Supplementary Table S2. Multivariable Logistic Regression Analysis Between S4-Phase Acoustic Signal Intensity and Echocardiographic Parameters Using Guideline-Based Cutoff Values

| Independent variable | Dependent variable | Log odds coefficient | SE | p-value |
|---|---|---|---|---|
| 4LSB level_2_area | septal E/e' ≥8 | 1.185 | 0.51 | 0.020 |
| | septal e' <7 | 2.10 | 0.77 | 0.0062 |
| | lateral e' <10 | 1.96 | 0.92 | 0.033 |
| | E/A >0.8 | -1.13 | 0.58 | 0.054 |
| 4LSB level_3_area | septal e' <7 | 5.44 | 1.93 | 0.0049 |
| | lateral e' <10 | 2.49 | 1.52 | 0.10 |
| | E/A >0.8 | -2.39 | 1.39 | 0.086 |
| 4LSB level_4_area | septal e' <7 | 10.37 | 5.25 | 0.048 |
| | E/A >0.8 | -8.79 | 5.52 | 0.11 |
| 5LMCL level_2_area | E/A >0.8 | -1.13 | 0.58 | 0.054 |
| 5LMCL level_3_area | E/A >0.8 | -2.39 | 1.39 | 0.086 |

Multivariable logistic regression analyses were conducted using heart sound level area and established contributors to diastolic dysfunction, including age, systolic blood pressure, history of coronary artery disease (CAD), HbA1c, and interventricular septal thickness, as independent variables. Dichotomized transthoracic echocardiographic (TTE) parameters, defined by guideline-recommended cutoff values, served as dependent variables. Only cases with FDR-q value <0.005 are presented.
4LSB, fourth left sternal border; 5LMCL, fifth left midclavicular line; AIC, Akaike Information Criterion; CAD, coronary artery disease; DcT, deceleration time; E/A, ratio of peak early diastolic (E) to peak atrial systolic (A) transmitral flow velocities; E/e′, ratio of transmitral E-wave velocity to mitral annular e′ velocity measured by tissue Doppler imaging; EF, left ventricular ejection fraction; IVS, interventricular septal thickness; LLR-p-value, Log-Likelihood Ratio p-value; LAD, left atrial diameter; LVDd, left ventricular diastolic diameter; R2, Nagelkerke $R^2$; sBP, systolic blood pressure; SE, standard error; TTE, transthoracic echocardiography.
